# Recent Advances in Research on Inhibitory Effects of Seaweed Extracts Against Parasites

**DOI:** 10.3390/md23040171

**Published:** 2025-04-16

**Authors:** Wenbing Cheng, Xiangyang Yang, Dengfeng Yang, Ting Zhang, Liguang Tian, Jiahao Dao, Zheng Feng, Wei Hu

**Affiliations:** 1 State Key Laboratory of Reproductive Regulation and Breeding of Grassland Livestock, Inner Mongolia Engineering Technology Research Center of Germplasm Resources Conservation and Utilization, School of Life Sciences, Inner Mongolia University, Yuquan District, Hohhot 010000, China; chengwenbing2025@163.com (W.C.); y2990892727@163.com (X.Y.); daoi333@163.com (J.D.); huw@imu.edu.cn (W.H.); 2 Guangxi Key Laboratory of Marine Natural Products and Combinatorial Biosynthesis Chemistry, Guangxi Beibu Gulf Marine Research Center, Guangxi Academy of Sciences, Daling Road No. 98, Nanning 530007, China; dengfengyang@163.com; 3 National Health Commission Key Laboratory of Echinococcosis Prevention and Control, Xizang Center for Disease Control and Prevention, Lhasa 850000, China; 4 National Institute of Parasitic Diseases, Chinese Center for Disease Control and Prevention (Chinese Center for Tropical Diseases Research), NHC Key Laboratory of Parasite and Vector Biology, WHO Collaborating Center for Tropical Diseases, National Center for International Research on Tropical Diseases, 207 Ruijin 2 Road, Shanghai 200025, China; tianlg@nipd.chinacdc.cn (L.T.); zfeng0909@163.com (Z.F.); 5 Laboratory of Diagnosis and Treatment of Zoonotic Parasitic Diseases, National Key Laboratory of Severe Diagnosis and Treatment of Zoonotic Infectious Diseases, College of Veterinary Medicine, Jilin University, Lvyuan District, Changchun 130062, China

**Keywords:** parasite, drug resistance, natural product, ocean, terpenoids, algae, *Plasmodium*, *Leishmania*, *Trypanosome*

## Abstract

Parasitic diseases pose a serious threat to the health of humans and the steady development of livestock husbandry. Although there are certain drug-based treatment methods, with the widespread application of drugs, various parasites are gradually developing drug resistance. Natural products are highly favored by researchers due to their characteristics such as low toxicity, multi-target effects, and low risk of drug resistance. The ocean, as the largest treasure trove of biological resources on Earth, has a special ecosystem (high pressure, high salt, and low oxygen). This enables marine organisms to develop a large number of unique structures during their survival competition. So far, a variety of compounds, such as terpenoids, have been isolated from the algae. As potential drugs, these compounds have certain curative effects on various diseases, including tumors, parasitic diseases, Alzheimer’s disease, and tuberculosis. This paper systematically reviews and analyzes the current advances in research on the antiparasite effects of seaweed extracts. The primary objective of this research is to formulate a conceptual foundation for marine pharmaceutical exploration, focusing on the creation of innovative marine-based medicinal compounds to overcome the emerging problem of parasite resistance to conventional treatments.

## 1. Introduction

Parasitic diseases not only hinder the healthy growth of livestock and pose a serious threat to the safety of herdsmen’s lives but also exert significant negative impacts on the economy. Despite the diverse types of parasitic diseases, the currently available therapeutic drugs are relatively scarce and face challenges of drug resistance, exacerbating the difficulty of treatment. The unique marine environment has drawn significant attention from researchers. In recent years, marine biological extracts, particularly those from seaweed, have been intensively studied for their potential against three major parasitic diseases: malaria, leishmaniasis, and trypanosomiasis. Among marine organisms, seaweed is relatively easy to obtain, manipulate in experiments, and extract compounds from. In light of this, the subsequent sections will provide a comprehensive overview of the disease burden, clinical manifestations, and additional pertinent details related to these three parasitic infections. Of course, while these three are the parasitic diseases, our literature analysis will also delve into various other parasite species.

Malaria, caused by protozoan parasites of the genus *Plasmodium*, is widely prevalent worldwide and constitutes a significant public health burden [1]. In 2023, the global incidence of malaria was estimated at 263 million cases, with approximately 597,000 fatalities attributed to the disease. The African region bore the overwhelming burden of malaria in 2023, representing approximately 94% of global cases and 95% of worldwide fatalities associated with the disease. In 2023, children under the age of five were particularly vulnerable to severe complications from malaria, representing nearly 76% of all malaria-related deaths in the WHO African Region (https://www.who.int/home/search-results?indexCatalogue=genericsearchindex1&searchQuery=Malaria&wordsMode=AnyWord) (accessed on 11 December 2024). Artemisinin stands as the most extensively utilized compound in the fight against malaria. Approximately a decade ago, resistance to artemisinin began to surface in Southeast Asia. In western Cambodia, which is located 800 km from the northwestern border of Thailand, the treatment failure rates for artesunate and mefloquine have been on the rise. Consequently, the development of novel drugs is imperative to counteract the threat posed by resistance to antimalarial agents [2].

Leishmaniasis is a disease caused by protozoan parasites belonging to the genus *Leishmania* [3]. Leishmanias can continue to spread among humans and animals, posing a serious threat to public health security [4]. Cutaneous leishmaniasis (CL), which is the predominant form of leishmaniasis, primarily induces ulcerative skin lesions on the body’s exposed areas. Approximately 95% of CL cases are concentrated in regions such as the Americas, the Mediterranean basin, the Middle East, and Central Asia. Globally, the annual incidence of new cases of CL is estimated to range from 600,000 to 1 million (https://www.who.int/home/search-results?indexCatalogue=genericsearchindex1&searchQuery=Leishmaniasis&wordsMode=AnyWord) (accessed on 12 January 2023). Antimonials, particularly sodium stibogluconate (SSG), were historically first-line treatments for leishmaniasis. However, due to widespread resistance in the Indian Subcontinent (ISC), SSG was largely discontinued and replaced by miltefosine (MIL). Yet, MIL efficacy has declined within a decade of its introduction [5].

Trypanosomiasis is a disease caused by parasites of the genus *Trypanosoma.* The ongoing global spread of trypanosomiasis poses a severe challenge to public health security [6]. There are two main forms of trypanosomiasis: African trypanosomiasis and American trypanosomiasis. African trypanosomiasis poses a significantly higher fatality risk compared to Chagas disease (American trypanosomiasis). African trypanosomiasis, a generally fatal disease if left untreated, is endemic in sub-Saharan Africa, with an estimated at-risk population of 55 million individuals during the 2016–2020 period (https://www.who.int/home/search-results?indexCatalogue=genericsearchindex1&searchQuery=Trypanosomiasis&wordsMode=AnyWord) (accessed on 2 May 2023). Eflornithine has emerged as the first-line treatment for African trypanosomiasis. However, eflornithine monotherapy presents clinical challenges, including an intensive administration regimen and prolonged treatment duration. Notably, laboratory studies demonstrate that resistance to eflornithine can be readily induced through TbAAT6 gene deletion or mutation [7].

Marine ecosystems, renowned for their unparalleled biodiversity and complex chemical profiles, serve as a significant source of bioactive pharmaceutical compounds. To date, 17 marine-inspired therapeutic agents have received clinical approval, while 28 candidate drugs, including cytarabine, eribulin, marizomib, plitidepsin, trabectedin, adcetris, zalypsis, and the largazole-based OKI-179, are progressing through various phases of clinical development [8]. Natural products derived from marine algae exhibit powerful and extensive bioactivities, including antibacterial, antiviral, antiparasitic, antituberculosis, antimycobacterial, antioxidant, anticoagulant, and anti-inflammatory functions [9]. At present, the problem of malaria resistance to artemisinin has drawn deep concern from the World Health Organization. Parasitic diseases not only face the challenging issue of drug resistance that is difficult to overcome but also have limited options for treatment drugs. More severely, the existing treatment drugs also have the drawbacks of low absorption efficiency and high toxicity. Consequently, the urgency to discover novel therapeutic agents has escalated. Emerging studies have underscored the significance of oceanic environments as essential repositories of pharmacologically relevant natural substances, exhibiting notable inhibitory activity against parasites, bacteria, and other pathogens that impact humans and animals. This makes the marine environment a promising source for the development of antiparasitic drugs. In view of this, we have systematically sorted out and summarized the drugs for treating parasites with seaweed extracts, aiming to deeply explore marine resources, solve the problem of parasite resistance, and establish a robust scientific foundation for therapeutic interventions against parasitic infections.

## 2. Retrieval Strategy and Results

### 2.1. Methods

#### 2.1.1. Study Design and Eligibility Criteria

In compliance with the Preferred Reporting Items for Systematic Reviews and Meta-Analyses (PRISMA) criteria, this review maintains rigorous adherence to standardized reporting protocols. In this review, the research time range is set from 2000 to 2024. During this period, all articles published in English that reported the biological activity of seaweed extracts against parasites such as *Leishmania parasites*, *Trypanosoma cruzi*, and *Plasmodium* were considered. Only articles that elaborate on the isolation and discovery process of seaweed extracts with antiparasitic biological activity will be officially included in the scope of this review.

#### 2.1.2. Search Strategy

In order to comprehensively and accurately obtain relevant literature, the following search strategy is formulated to search for all articles related to the antiparasitic research of seaweed extracts published in English between 2000 and 2024. Use Boolean operators to combine the following terms and search in the PubMed database: (((((((((((((Antiparasitic [Title/Abstract]) OR (Parasiticide [Title/Abstract])) OR (Communicable Disease [Title/Abstract])) OR (Helminth [Title/Abstract])) OR (Parasite [Title/Abstract])) OR (Worm [Title/Abstract])) OR (Zoonoses [Title/Abstract])) OR (antileishmanial [Title/Abstract])) OR (antiplasmodial [Title/Abstract])) OR (antitrypanosomal [Title/Abstract])))) AND (((((((((Marine Natural Products [MeSH Terms]) OR (Marine drugs [Title/Abstract])) OR (Natural compounds [Title/Abstract])) OR (seaweed [Title/Abstract])) OR (algae [Title/Abstract])) OR (Marine plants [Title/Abstract])) OR (Marine natural products [Title/Abstract])) OR (Marine drugs [Title/Abstract])) OR (Natural compounds [Title/Abstract]))).

#### 2.1.3. Research Selection and Data Extraction

Conduct a comprehensive literature search in the PubMed database according to the search strategy. Upon retrieving a substantial number of search results, assess the titles and abstracts of the retrieved articles, and eliminate those with redundant content or that are irrelevant to the focus of this review. For the articles that were retained after screening, the full-text content was downloaded and further evaluated in depth and comprehensively. After a series of rigorous evaluations, relevant data for the articles included in this review were ultimately extracted.

### 2.2. Results

#### 2.2.1. Study Selection

**Figure 1:**
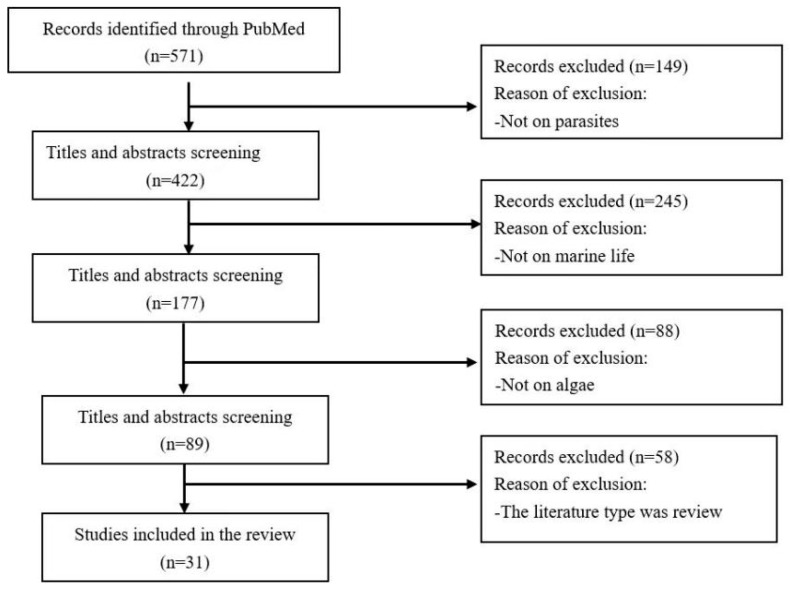
Screening procedure for study identification in this analysis.

After conducting extensive searches in the PubMed database, a preliminary search yielded 571 published articles. After removing duplicates, the titles and abstracts of the articles were screened, and 422 articles unrelated to the topic of this review were excluded, resulting in 89 articles. After screening and evaluating the full texts of 89 articles, this review ultimately used 31 articles that met the criteria (Figure 1).

#### 2.2.2. Characteristics Included in the Study

The studies included in this review revolve around extracts isolated or identified from seaweed, which exhibit significant antiparasitic activity against *Trypanosoma*, *Leishmania*, and *Plasmodium*. In the research on the antiparasitic effects of seaweed extracts, there are mainly red algae, green algae, and brown algae. These seaweed extracts not only demonstrate significant potential in antiparasitic activity but also lay the foundation for further exploration of their antiparasitic mechanisms and novel antiparasitic drug discovery.

## 3. Red Algae

### 3.1. Terpenoids Derived from Red Algae

**Figure 2:**
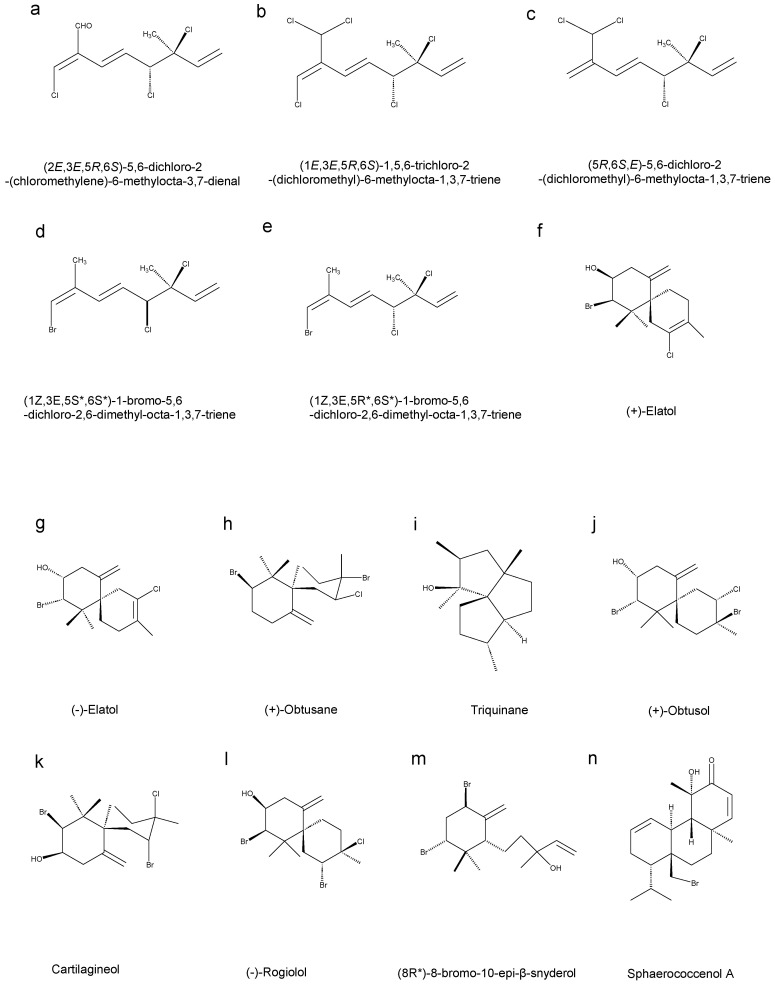
Terpenoid compounds in red algae. (**a**) (2E,3E,5R,6S)-5,6-dichloro-2-(chloromethylene)-6-methylocta-3,7-dienal, (**b**) (1E,3E,5R,6S)-1,5,6-trichloro-2-(dichloromethyl)-6-methylocta-1,3,7-triene, (**c**) (5R,6S,E)-5,6-dichloro-2-(dichloromethyl)-6-methylocta-1,3,7-triene, (**d**) (1Z,3E,5S*,6S*)-1-bromo-5,6-dichloro-2,6-dimethyl-octa-1,3,7-triene, (**e**) (1Z,3E,5R*,6S*)-1-bromo-5,6-dichloro-2,6-dimethyl-octa-1,3,7-triene, (**f**) (+)-Elatol, (**g**) (-)-Elatol, (**h**) (+)-Obtusane, (**i**) Triquinane, (**j**) (+)-Obtusol, (**k**) Cartilagineol, (**l**) (-)-Rogiolol, (**m**) (8R*)-8-bromo-10-epi-β-snyderol, (**n**) Sphaerococcenol A.

Afolayan et al. extracted antimalarial compounds (**1**–**3**) (Figure 2a–c), and two novel compounds (**4** and **5**) (Figure 2d,e) from the red alga *Plocamium cornutum*, with pharmacological evaluation showing that compounds 2 (IC_50_ values of 16 μM) and 3 (IC_50_ values of 17 μM) possessed the highest potency, while compound 1 demonstrated weaker activity (IC_50_ values of 27 μM), and compounds **4** and **5** exhibited relatively low activity (IC_50_ values of 230 μM and 210 μM) [10].

Elatol, a halogenated sesquiterpene alcohol extracted from the Brazilian red seaweed *Laurencia dendroidea*, has shown inhibition of parasitic activity against epimastigotes, trypomastigotes, and amastigotes (IC_50_ values of 45.4 μM, 1.38 μM, and 1.01 μM, respectively.) Additionally, when considering the effect of elatol on red blood cells, its CC_50_ value was measured as 27.0 μM [11]. After a 72 h treatment, elatol exhibited significant antileishmanial activity against *L. amazonensis* with IC_50_ values of 4.0 µM for the promastigote stage and 0.45 µM for the intracellular amastigote stage. The cytotoxicity of this compound to macrophages was assayed, and its 50% cytotoxic concentration (CC_50_) was determined to be 1.4 µM [12].

Chamigrane-type sesquiterpenoids, derived from the red alga *Laurencia dendroidea*, hold promise as lead compounds for developing agents against *Naegleria fowleri*. This study discovered two new compounds, (+)-elatol and (−)-elatol, which exhibited opposite specific rotations of $\left[\alpha_{D}^{20}\right]$ + 80 (c 0.20, CHCl3) and $\left[\alpha_{D}^{20}\right]$ − 80 (c 0.20, CHCl3), confirming their enantiomers (Figure 2f,g). The compounds are chamigrane-type sesquiterpenoids featuring a helical [5.5] undecane skeleton with a chiral quaternary carbon (C-6) connected to a helical structure. (+)-Elatol demonstrates the greatest activity against *N. fowleri trophozoites*, exhibiting inhibitory concentrations of 1.08 μM (ATCC 30808™) and 1.14 μM (ATCC 30215™). Conversely, its enantiomer, (−)-Elatol, exhibits significantly lower activity, with IC_50_ values of 36.77 μM and 38.03 μM for the strains, which are roughly 34-fold higher than those of (+)-Elatol. Moreover, the IC_50_ value of (+)-Elatol against the cysts of the *Naegleria fowleri* ATCC 30808™ strain is 1.14 μM; at this concentration, it does not exhibit cytotoxicity [13].

Da et al. investigated bioactive crude extracts from the red alga *Laurencia dendroidea* collected along the southeastern Brazilian coast, identifieing five sesquiterpenes: obtusane, a triquinane derivative, (-)-elatol, obtusol, and cartilagineol (Figure 2g–k). Bioassays of the triquinane derivatives elatol and obtusol against *L. amazonensis*, revealed potent inhibitory effects against both promastigote (IC_50_ values of 43.8, 6.2, and 9.7 µg/mL, respectively) and amastigote forms (IC_50_ values of 48.7 ± 3.7, 3.9, and 4.5 µg/mL, respectively) with cytotoxicity assessments demonstrated favorable safety profiles showing no significant toxicity to peritoneal macrophages or lymph node cells under the experimental parameters [14].

Three halogenated sesquiterpenes (Figure 2j–l) were extracted from the red algae *Laurencia dendroidea*, namely Elastin, Rogiolol, and Obtosol. After treating 96 adult *Schistosoma mansoni* with three halogenated compounds at a concentration of 50 µg/mL, experimental results revealed that Rogiolol treatment resulted in total couple separation at 24 h and achieved 90% worm lethality by 72 h. Obtusol caused a 20% mortality rate in female worms, with no effect on male survival. Conversely, elatol had no impact on worm survival and only slightly affected their motility. However, elatol resulted in a complete (100%) separation of worm pairs and fully suppressed oviposition [15].

The red alga *Laurencia obtusa* yielded the isolation of (8R*)-8-bromo-10-epi-β-snyderoland (Figure 2m) as a secondary metabolite. This compound demonstrated moderate bioactivity. Against the D6, its IC_50_ values are 2700 ng/mL, and against the W2, the IC_50_ values are 4000 ng/mL. As a control, artemisinin exhibited IC_50_ values of 2.8 ng/mL for the D6 strain and 1.1 ng/mL for the W2 strain [16].

Collected from Moroccan Atlantic coastal waters, *Sphaerococcus coronopifolius* yielded Sphaerococcenol A (Figure 2n), a bromo-diterpene that showed significant antiplasmodial activity (IC_50_ values of 1 µM) against the chloroquine-resistant FCB1 strain of *P. falciparum* [17].

**Figure 3:**
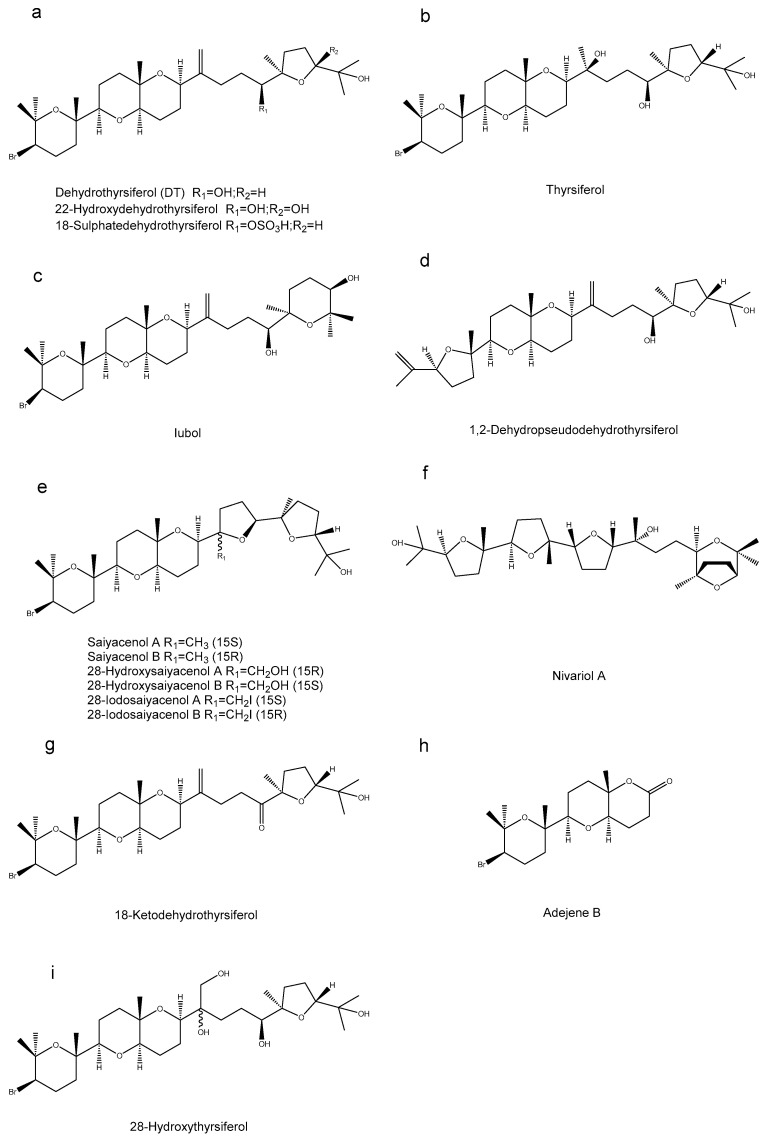
Terpenoid compounds in red algae. (**a**) Dehydrothyrsiferol (DT) R_1_=OH;R_2_=H, 22-Hydroxydehydrothyrsiferol R_1_=OH;R_2_=OH, 18-Sulphatedehydrothyrsiferol R_1_=OSO_3_H;R_2_=H, (**b**) Thyrsiferol, (**c**) Iubol, (**d**) 1,2-Dehydropseudodehydrothyrsiferol, (**e**) Saiyacenol A R_1_=CH_3_ (15S), Saiyacenol B R_1_=CH_3_ (15R), 28-Hydroxysaiyacenol A R_1_=CH_2_OH (15R), 28-Hydroxysaiyacenol B R_1_=CH_2_OH (15S), 28-Iodosaiyacenol A R_1_=CH_2_I (15S), 28-Iodosaiyacenol B R_1_=CH_2_I (15R), (**f**) Nivariol A, (**g**) 18-Ketodehydrothyrsiferol, (**h**) Adejene B, (**i**) 28-Hydroxythyrsiferol.

In the marine environment, polyether triterpenoids (Figure 3a–i) were either isolated or semi-synthesized from the red alga *Laurencia viridis*. Among the tested compounds, six demonstrated efficacy against *L. amazonensis*. The IC_50_ values of these six compounds ranged from 5.40 ± 0.13 µM for 28-iodosaiyacenol B to 20.39 ± 2.90 µM for 28-iodosaiyacenol A. Moreover, four of the polyether triterpenoids demonstrated the ability to inhibit *L. donovani*. Specifically, iubol exhibited an IC_50_ values of 18.08 ± 2.62 μM; 28-iodosaiyacenol A had an IC_50_ values of 33.90 ± 1.74 μM; dehydrothyrsiferol showed an IC_50_ values of 28.26 ± 1.74 μM; and 18-ketodehydrothyrsiferol presented an IC_50_ values of 46.45 ± 2.26 μM. Conversely, *T. cruzi* proved to be the most resistant parasite among those tested, as only four of the polyether triterpenoids had activity against it. The four active compounds identified were iubol (IC_50_ values of 9.20 ± 1.16 µM), DT (IC_50_ values of 9.45 ± 0.46 µM), 18-ketodehydrothyrsiferol (IC_50_ values of 11.02 ± 2.60 µM), and saiyacenol-A (IC_50_ values of 13.75 ± 2.28 µM) [18].

Sixteen oxidized squalene derivatives (Figure 3a–i) were isolated from the red alga, and their antiacanthamoeba activities were evaluated. All tested compounds exhibited inhibitory effects against the trophozoites of *Acanthamoeba castellanii Neff*. The pharmacological evaluation revealed distinct activity profiles among the tested compounds, with Iubol exhibiting the most pronounced efficacy (IC_50_ values of 5.30 ± 0.87 µM), demonstrating comparable potency to chlorhexidine. Following Iubol, dehydrothyrsiferol (DT) displayed substantial biological activity, yielding an IC_50_ value of 12.83 ± 1.38 μM. The IC_50_ values for 22-Hydroxydehydrothyrsiferol and thyrsiferol were found to be 13.97 ± 1.57 µM and 17.00 ± 4.57 µM, indicating that their activities were comparable. Additionally, DT displayed cysticidal activity against *Acanthamoeba castellanii* cysts (IC_50_ values of 39.27 ± 0.14 μM). The IC_50_ values for DT against *A. griffini* and *A. polyphaga* were 56.66 ± 2.09 μM and 73.44 ± 4.02 μM. Iubol demonstrated the highest toxicity towards mouse macrophages J774A.1, with a CC_50_ value of 7.72 ± 0.22 μM, while DT exhibited lower toxicity (CC_50_ = 28.77 ± 3.10 μM) [19].

Among the terpenoids extracted from red algae, there are five monoterpenoids, nine sesquiterpenoids, one diterpenoid and six triterpenoids. Seven of these terpenoids can inhibit *Plasmodium*, 10 show inhibitory effects on *L. amazonensis*, four compounds can inhibit *L. donovani*, four can inhibit *T. cruzi*, three can inhibit *S. mansoni*, and two can inhibit the trophozoites of *Naegleria fowleri*. The IC_50_ values of (8R*)-8-bromo-10-epi-β-snyderol against the D6 and W2 clones of *Plasmodium* are 2700 ng/mL and 4000 ng/mL. The compound also displays potent antimalarial properties, showing an IC_50_ value of 1 µmol against the chloroquine-resistant *P. falciparum* FCB1 strain. Rogiolol has the most significant impact on *S. mansoni*. After treatment with 50 μg/mL of Rogiolol for 72 h, the mortality rate of *S. mansoni* can reach 90%. Elatol and obtusol demonstrated inhibitory effects on promastigotes. The IC_50_ values for elatol and obtusol against promastigotes were 6.2 μg/mL and 9.7 μg/mL. Additionally, these compounds also showed inhibitory activity against amastigotes, with elatol having an IC_50_ value of 3.9 μg/mL and obtusol an IC_50_ value of 4.5 μg/mL. Currently, among the terpenoids from seaweed extracts, those acting on *Leishmania* have been studied the most, and elatol is the most investigated in terms of its inhibitory effect on *Leishmania*. In the next step, elatol can be applied to protozoa such as *Plasmodium*, *Trypanosoma* and *Cryptosporidium* to explore whether it also has certain inhibitory effects on other parasites.

### 3.2. Other Components Derived from Red Algae

Teixeira et al. assessed various extracts from the red alga *Plocamium brasiliense* against *T. cruzi*, finding that for intracellular amastigotes, the crude extract has an IC_50_ value of 7.1 μg/mL and mixture F has a value of 4.9 μg/mL, while for trypomastigotes, the crude extract has an IC_50_ value of 10.8 μg/mL and mixture F has an IC_50_ value of 20.3 μg/mL [20].

This investigation examined the antibacterial activities of both crude extracts and chromatographic fractions derived from *Asparagopsis taxiformis* and *Asparagopsis armata*, two red algal species sampled from the coastal waters of the Messina Strait, Italy. The investigation focused on their effectiveness against *Leishmania*. Notably, the hexane and dichloromethane crude extracts of *Asparagopsis taxiformis* exhibited substantial inhibitory effects on *Leishmania*. In detail, the half maximal inhibitory concentration (IC_50_) of the hexane crude extract of *A. taxiformis* was found to be 17.00 μg/mL, while the inhibitory concentration needed to achieve 90% inhibition (IC_90_) was determined to be 33.00 μg/mL [21].

The ethanol-based crude extracts derived from the marine macroalga *Asparagopsis taxiformis* demonstrated significant antileishmanial activity against *Leishmania infantum* parasites. When the concentration of the extracts reached 40 μg/mL, complete (100%) mortality of the parasite was achieved. Cytotoxicity assessment revealed LD_50_ values of 25 μg/mL and 9 μg/mL, killing promastigote and amastigote forms, while demonstrating no cytotoxic effects on DH82 and Vero mammalian cell lines [22].

A comprehensive screening study performed by Allmendinger et al. evaluated crude extracts from 23 red algal species collected from coastal regions of England and Ireland identifying *Corallina officinalis* and *Ceramium virgatum* as particularly effective activity against *T. brucei* rhodesiense with IC_50_ values of 4.8 μg/mL and 5.4 μg/mL, respectively, while all algal extracts showed activity against *T. brucei* rhodesiense and exception for *Porphyra leucosticta*, displayed leishmanicidal efficacy, with IC_50_ concentrations ranging from 16.5 μg/mL to 85.6 μg/mL [23].

The IC_50_ value of the hexane extract of the red alga *Peyssonelia squamaria* against *Acanthamoeba castellanii Neff* was 134.6 ± 0.7 µg/mL, that of the ethyl acetate extract was 52.3 ± 1.8 µg/mL, and that of the methanol extract was 76.1 ± 0.9 µg/mL [24].

## 4. Brown Algae

### 4.1. Terpenoids Derived from Brown Algae

**Figure 4:**
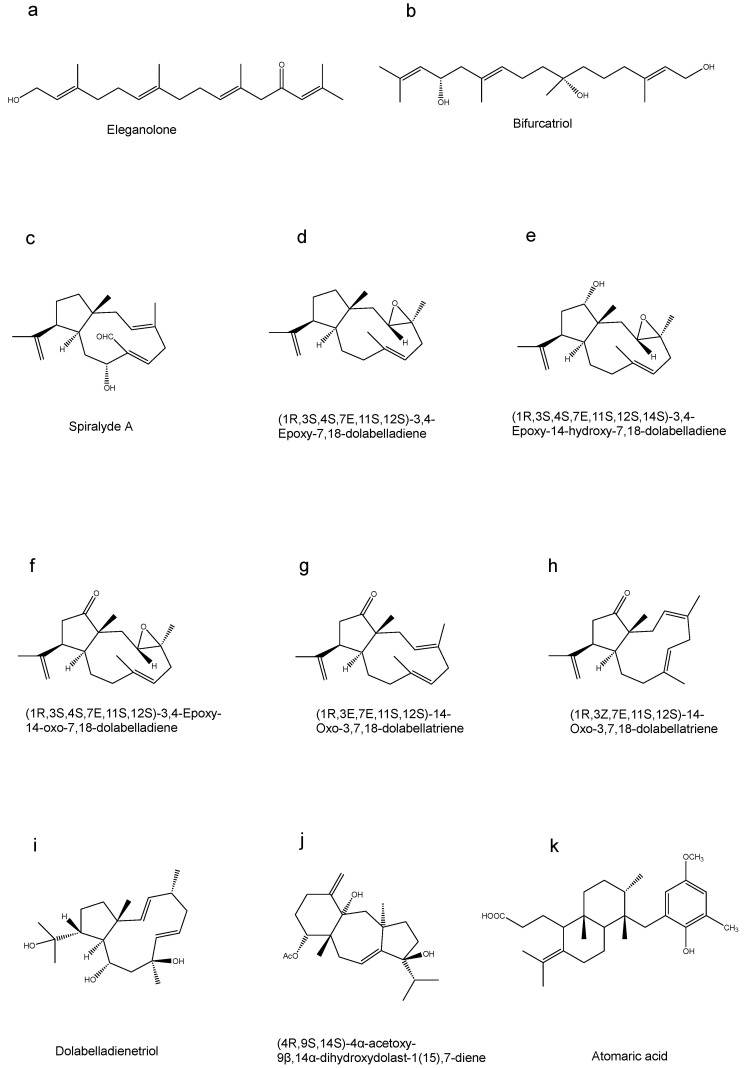
Terpenoid compounds in brown algae. (**a**) Eleganolone, (**b**) Bifurcatriol, (**c**) Spiralyde A, (**d**) (1R,3S,4S,7E,11S,12S)-3,4-Epoxy-7,18-dolabelladiene, (**e**) (1R,3S,4S,7E,11S,12S,14S)-3,4-Epoxy-14-hydroxy-7,18-dolabelladiene, (**f**) (1R,3S,4S,7E,11S,12S)-3,4-Epoxy-14-oxo-7,18-dolabelladiene, (**g**) (1R,3E,7E,11S,12S)-14-Oxo-3,7,18-dolabellatriene, (**h**) (1R,3Z,7E,11S,12S)-14-Oxo-3,7,18-dolabellatriene, (**i**) Dolabelladienetriol, (**j**) (4R,9S,14S)-4α-acetoxy-9β,14α-dihydroxydolast-1(15),7-diene, (**k**) Atomaric acid.

The ethyl acetate fraction of French-collected *Bifurcaria bifurcatriol* yielded eleganolone (Figure 4a), a linear diterpenoid compound. This metabolite exhibited differential antiparasitic activity, displaying moderate efficacy against *T. brucei* (IC_50_ value of 13.7 µg/mL) while showing enhanced selectivity against *P. falciparum* (IC_50_ value of 7.9 µg/mL) [25].

The Irish marine alga *Bifurcaria bifurcata* has yielded biurcatriol (Figure 4b), a bioactive compound displaying varying antiparasitic efficacy. This metabolite demonstrated IC_50_ values of 11.8 µg/mL (*T.brucei rhodesiense*), 47.8 µg/mL (*T. cruzi*), 18.8 µg/mL *(L. donovani*), and 0.65 µg/mL (*P. falciparum*), respectively. Notably, the compound showed favorable selectivity with low cytotoxicity in L6 cells (IC_50_ value of 56.6 µg/mL) [26].

The brown seaweed *Dictyota spiralis* yielded spiralyde A, a novel meta-dolabellane aldehyde derivative, along with five known diterpenoid compounds (Figure 4c–h). Among these secondary metabolites, spiralyde A exhibited superior antiparasitic efficacy, showing IC_50_ values of 5.62 ± 2.48 μM against *T. cruzi* and 15.47 ± 0.26 μM against *Leishmania* species. The findings demonstrated efficacy similar to that of benznidazole, a reference drug used to treat *T. cruzi* infections (IC_50_ values of 6.94 ± 1.94 µM). The IC_50_ values of (1R,3S,4S,7E,11S,12S)-3,4-Epoxy-7,18-dolabelladiene against *T. cruzi* and *L. amazonensis* were 35.29 ± 4.09 μM and 36.81 ± 5.20 μM. Other compounds exhibited no significant activity at a concentration of 100 μM [27].

Dolabelladienetriol (Figure 4i), a diterpenoid metabolite obtained from the Brazilian marine alga *Dictyota pfaffii* sampled along the northeastern coastal region, demonstrates significant antileishmanial properties. After a 72 h exposure period at concentrations ranging from 50 to 100 μM, the growth of proflagellate was inhibited by 84% and 95.5%. The compound exhibited a dose-dependent inhibitory effect on intracellular amastigotes. After 24 h exposure to 50 μM and 100 μM concentrations, the growth inhibition was reduced by 56% and 61%. Notably, these treatments did not exhibit significant toxicity towards host cells [28].

4α-Acetoxy-9β,14α-dihydroxydolasta-1(15),7-diene (Figure 4j), a diterpenoid metabolite derived from *Canistrocarpus cervicornis* collected along the Brazilian coast, demonstrated dose-dependent antileishmanial efficacy against promastigote forms, with an IC_50_ value of 2 μg/mL. The IC_50_ values for amastigotes and intracellular amastigotes were 12 μg/mL and 4 μg/mL after 24 h of treatment, resulting in a significant reduction in the survival index of amastigotes. The selectivity index (SI) of the compound is 93.0, indicating that its toxicity to macrophages is 93.0-fold lower than that to protozoa [29].

The meroditerpenoid compound atomaric acid (Figure 4k) was obtained from the marine macroalga *Stypopodium zonale*. The compound for the intracellular amastigotes of *T. cruzi* IC_50_ values of 2.4 μg/mL/5.42 μM, SI = 16.75, but was lower than that of Benznidazole (positive control) IC_50_ values of 1 μg/mL/2 μM. For the intracellular amastigotes of *T. cruzi* Y strain, atomaric acid also presented relatively high activity IC_50_ values of 1.77 μg/mL/4 μM, which was lower than that of Benznidazole (IC_50_ < 0.5 μg/mL/1 μM). Furthermore, atomaric acid shows moderate toxicity to Vero cells (CC_50_ = 40.2 μg/mL/90.8 μM) [20].

Among the terpenoid extracts of brown algae, there are six diterpenoid compounds and only one meroterpenoid compound. There are four compounds that have inhibitory effects against Trypanosomes, two that act against malaria parasites, and as many as five that have inhibitory effects against *Leishmania*. It is particularly worth mentioning that Spiralyde A is on a par with the new drug benznidazole in terms of its efficacy in treating *Trypanosomes*. Regarding the monoterpenoids and sesquiterpenoids in brown algal extracts, there is relatively little relevant research at present. Whether it is due to the difficulty of extraction resulting in unsuccessful extraction or because their biological activities or therapeutic effects are not significant, further in-depth research and exploration are needed to determine.

### 4.2. Extract Mixture from Brown Algae

Extracts derived from the French brown alga *Bifurcaria bifurcata* exhibited substantial anthelmintic efficacy in assays evaluating larval development of *Heligmosomoides polygyrus bakeri*. Complete inhibition of *H. polygyrus* bakeri larval maturation (L1/L2 to L3) was achieved using 5 mg/mL water-based extracts prepared by cold and hot extraction methodologies. The IC_50_ values obtained from the cold and hot extraction methods were 0.68 ± 0.02 mg/mL and 0.97 ± 0.03 mg/mL. A 5 mg/mL aqueous extract resulted in a hatching inhibition rate of 28% for eggs and a mortality rate of 67% for newly hatched larvae and exhibited significant inhibitory effects on adult nematode activity. Complete mortality was observed within 24 h at a 5 mg/mL concentration. The viability rates declined to 10% following a 72 h exposure at 1 mg/mL and showed a further reduction to 30% after 144 h at 0.5 mg/mL concentration [30].

From the brown seaweed *Ccharina latissima*, four biologically active metabolites were purified. The Danish-derived sample SW1, prepared through methanol/water extraction, showed limited efficacy in suppressing egg hatching. In marked distinction, sample SW2 collected from the Faroe Islands demonstrated contrasting properties demonstrated a potent inhibitory effect on egg hatching, with a statistically significant difference (*p* < 0.05). Additionally, the two samples, SW3 and SW4, which were derived from dichloromethane and methanol, also significantly suppressed egg hatching. However, their inhibitory effects were not as pronounced as those of SW2. Collectively, the findings from these experiments clearly indicate that cinnamaldehyde, along with the compounds derived from the seaweed, possesses antiparasitic properties specifically targeting the egg stage of *Uncinaria stenocephala* [31].

The marine macroalga *Padina avonica* was sourced from Egyptian Red Sea coastal waters, and following taxonomic identification, a 70% ethanol extract was prepared. Six extracts at varying concentrations (5, 25, 50, 75, 100, and 160 µg/mL) were prepared to interact with the trophoblasts and cysts of *Acanthamoeba castellanii* over different incubation periods. The viability of trophoblasts and cysts was significantly reduced following treatment with varying concentrations of the extracts, with complete inhibition observed at higher concentrations. The inhibition rate increased in a concentration- and incubation-time-dependent manner, and the inhibitory effect on trophoblasts was consistently greater than that on cysts at different incubation times. Applied after 24 h, the IC_50_ values for trophoblasts and cysts were determined to be 4.56 µg/mL and 4.89 µg/mL [32].

Bioactive compounds with antiparasitic properties were isolated from Albanian marine macroalgae, specifically *Sargassum vulgare* and *Cladostephus hirsutus,* and the ethyl acetate fractions of both extracts demonstrated in vitro antitrypanosomal activity. The *S. vulgare* extract exhibited potent trypanocidal effects against *T*. *brucei*, displaying an IC_50_ value of 9.3 ± 4.9 μg/mL. *C. hirsutus*, however, showed limited antitrypanosome potential, exhibiting an IC_50_ value of 27.2 ± 5.0 μg/mL. At 50 μg/mL concentration, *S. vulgare* and *C. hirsutus* displayed inhibition rates of 95.5% and 89.7%. At a concentration of 10 μg/mL, neither compound exhibited significant antiplasmodial activity [33].

The dichloromethane/methanol (one to two) extract (DME) of *Dictyota mertensii* exhibits in vitro bioactivity against *L.amazonensis*, demonstrating dose-dependent inhibition of promastigote growth. The extract demonstrated an IC_50_ value of 71.6 ± 9.29 μg/mL, achieving complete growth inhibition (100%) at 250 μg/mL concentration. DME treatment demonstrated potent antileishmanial activity by inhibiting intracellular amastigotes in macrophage cultures, exhibiting an IC_50_ value of 81.4 ± 1.7 μg/mL following 18 h incubation. Furthermore, the compound showed favorable selectivity with a macrophage cytotoxicity CC_50_ value of 233.10 ± 25.5 μg/mL. The selectivity index (SI) for the promastigote form was 3.25, and for the amastigote form, it was 2.86 [34].

From the marine macroalga *Dictyota menstrualis*, a mixture of diterpenoid isomers, including pachydictyol A and isopachydictyol A, was isolated. This compound mixture demonstrated significant antileishmanial activity. with an EC_50_/24h value of 23.5 ± 15.4 μg/mL. In comparison with the control drug pentamidine, whose EC_50_/24h value was 7.6 ± 2.5 μg/mL, the activity of the mixture was inferior to that of pentamidine. The CC_50_/24 h value of the mixture on macrophages was 30.0 ± 7.0 μg/mL, and the SI value was 1.27 [35].

In vitro evaluation revealed that the ethyl acetate-soluble fraction from *B. bifurcata* displayed significant antiparasitic activity against *P. falciparum*, *T. cruzi*, and *L. donovani*. For Du’s sterile flagellar body, the IC_50_ values are 3.9 μg/mL. However, the extract demonstrated a low selectivity index of 1.6, suggesting its general toxicity [36].

This study examined the leishmanicidal activity and cellular toxicity of a bioactive extract obtained from *Bifurcaria bifurcata*, a brown seaweed species sampled from Morocco’s Atlantic shoreline. The research focused on assessing both the antiparasitic properties inhibiting *Leishmania* species and the potential cytotoxic impacts of the algal extract. Using a Soxhlet apparatus, sequential extraction was performed with progressively polar solvents (hexane → ether → chloroform → methanol), yielding four fractions designated as H, E, C, and M. Among these, fractions H, E, and C showed significant antileishmanial efficacy, with IC_50_ values of 46.83, 51.64, and 63.83 μg/mL, respectively. The methanol extract M was non-active. Extracts H, E and C are toxic to brine shrimp larvae, with the mortality rate increasing as the concentration rises. The LD_50_ values are 21.8 µg/mL, 40.46 µg/mL, and 37.05 µg/mL, respectively. Notably, the methanol extract (M) exhibited no cytotoxic effects [37].

The antileishmanial potential of *Bifurcaria bifurcata* was assessed through organic solvent extraction using hexane, diethyl ether, chloroform, and methanol. The prepared algal extracts exhibited potent antileishmanial properties, showing inhibitory concentrations (IC_50_) within the range of 46.83 to 63.83 µg/mL against *Leishmania* species. Cytotoxicity evaluation revealed an LD_50_ below 40.46 µg/mL, indicating potential therapeutic selectivity [38].

Against *Acanthamoeba castellanii* Neff, the IC_50_ values of the hexane extracts from the brown algae *Cladostephus spongiosum*, *Cystoseira sedoides*, *Dictyota spiralis*, *Padina pavonica*, and *Halopteris scoparia* were distinctly different. Specifically, they were 88.9 ± 0.1 µg/mL, 100.9 ± 3.5 µg/mL, 105.4 ± 2.0 µg/mL, 103.7 ± 4.6 µg/mL, and 84.0 ± 1.1 µg/mL, respectively. When it came to the ethyl acetate extracts of these same brown algae, their IC_50_ values against *Acanthamoeba castellanii Neff* were 54.9 ± 1.3 µg/mL for *Cladostephus spongiosum*, 86.0 ± 0.0 µg/mL for *Cystoseira sedoides*, 68.2 ± 5.2 µg/mL for *Dictyota spiralis*, 111.1 ± 0.6 µg/mL for *Padina pavonica*, and 105.7 ± 0.3 µg/mL for *Halopteris scoparia*. Moreover, the IC_50_ values of the methanol extracts of these brown algae against *Acanthamoeba castellanii Neff* were 91.3 ± 1.5 µg/mL, 83.6 ± 1.6 µg/mL, 64.3 ± 1.2 µg/mL, 91.9 ± 0.2 µg/mL, and 85.0 ± 0.1 µg/mL, respectively, for *C. spongiosum*, *C. sedoides*, *D. spiralis*, *P. pavonica*, and *H. scoparia* [24].

## 5. Green Algae

Six compounds were identified in the ethanol crude extract of *Halimeda macroloba*. Among these, 2,5-bis(6-iodo-10-methyltridecan-2-yl)-3,6-dimethylcyclohexa-2,5-diene-1,4-dione exhibited an IC_50_ value of 3.2 ± 0.23 µg/mL against *P. falciparum*, demonstrating excellent inhibitory activity, while 4,8,12-trimethylpentadecan-1-ol showed an IC_50_ value of 19.3 ± 0.51 µg/mL, indicating moderate inhibitory activity [39].

This study evaluated the antiparasitic properties of crude extracts from four British green algae species (*Cladophora rupestris*, *Codium fragile subspecies tomentosoides*, *Ulva intestinalis*, and *Ulva lactuca*) against various protozoan parasites. The extracts exhibited varying degrees of trypanocidal activity against *T. brucei* rhodesiense, with *C. rupestris* showing the highest potency (IC_50_ values of 3.7 μg/mL), followed by *C. fragile subspecies tomentosoides* (8.9 μg/mL), *U. intestinalis* (11 μg/mL), and *U. lactuca* (18 μg/mL). Against *T. cruzi*, only *U. lactuca* (IC_50_ values of 34.9 μg/mL) and *C. rupestris* (80.8 μg/mL) demonstrated moderate activity. All extracts displayed leishmanicidal effects against *L. donovani* amastigotes, with IC_50_ values ranging from 12.0 μg/mL (*U. lactuca*) to 20.2 μg/mL (*C. rupestris*). Importantly, none of the extracts showed cytotoxicity toward L6 cells, indicating selective antiprotozoal activity [40].

A comparative investigation of antitrichomonal properties was conducted on organic extracts obtained from 25 tropical marine macroalgae species harvested along the Yucatan Peninsula (Mexico). While metronidazole demonstrated potent activity with an IC_50_ value of 40 ng/mL, only two algal extracts exhibited significant inhibitory effects: *Udotea conglutinate* (Chlorophyta) with an IC_50_ value of 1660 ng/mL and *Lobophora variegata* (Phaeophyceae) showing an IC_50_ value of 1390 ng/mL [41].

The IC_50_ value of the hexane extract of the green alga *Ulva compressa* against *Acanthamoeba castellanii Neff* was 108 ± 0.1 µg/mL, that of the ethyl acetate extract was 84.2 ± 0.0 µg/mL, and that of the methanol extract was 54.4 ± 1.1 µg/mL [24].

## 6. Parasitic Diseases

In recent years, with the continuous advancement of marine drug research, natural polysaccharides extracted from seaweed (such as porphyran, alginate, carrageenan, etc.) have been experimentally confirmed to serve as efficient drug delivery carriers due to their unique physicochemical properties and biocompatibility. By constructing sustained-release systems such as nanoparticles, hydrogels, or microspheres, these polysaccharides can not only significantly enhance the targeted delivery efficiency of drugs in vivo but also effectively reduce drug toxicity to normal tissues through controlled release kinetics. Building on previous findings demonstrating the antiplasmodial, antileishmanial, and other antiparasitic activities of terpene compounds (e.g., sesquiterpenes, diterpene derivatives) and crude extracts with undefined chemical structures (e.g., seaweed ethanol extracts, polar solvent extracts), combined with the carrier advantages of marine algal polysaccharides, it is worth exploring whether the composite delivery systems formed by these two components can be further developed for the treatment of parasitic diseases.

### 6.1. Development of Seaweed Extract for Disease Delivery

Porphyran, a sulfated polysaccharide derived from the marine red macroalga *Porphyra haitanensis*, possesses antitumor activity. 5-fluorouracil is a frequently utilized antitumor medication, yet it exhibits considerable side effects. Wang and Zhang et al. have fabricated a low-molecular-weight porphyran (LP) complex loaded with LP-5Fu explored its antitumor and immunomodulatory activities in S180 tumor-transplanted mice and found that LP enhances antitumor effects through immune modulation rather than cytotoxicity, significantly enhancing the lymphocyte proliferation, restoring the levels of TNF-α and NO, and improving 5-Fu’s efficacy while mitigating its immunosuppression effects [42].

Curcumin, a hydrophobic polyphenol with diverse biological activities, suffers from poor water solubility and low bioavailability, which constrains its clinical application. κ-Carrageenan, a natural polysaccharide extracted from red algae, and the κ-carrageenan and curcumin form a complex via chemical interaction. The fabricated κ-Car-Cur microspheres exhibit a spongy morphology, are sensitive to the environmental pH, and possess a relatively high loading efficiency. The drug loading ratio of κ-Car-Cur was 8.4%, and the encapsulation efficiency was 73.6%. The in vitro release experiments demonstrated that, under acidic conditions at pH 5.0, the drug release rate was accelerated, achieving a cumulative release of up to 78%. In contrast, at pH 7.4, the cumulative release rate was only 43%. This indicates that κ-carrageenan can act as a potential carrier for drug delivery in the low pH environment of cancer cells [43].

Researchers developed a non-viral gene delivery system using ethanediamine-modified Porphyra yezoensis polysaccharide (Ed-PYP) as a carrier. The optimal formulation, with a 40:1 weight ratio of Ed-PYP to pABT plasmids (encoding Ascl1, Brn4, and Tcf3), demonstrated efficient gene delivery. Analysis showed peak expression of neural growth factors (NGF, BDNF, SHH) at 14 days post-transfection, accompanied by strong expression of neural markers (Nestin, GFAP, β-3tubulin, NF200, GAP43, MAP2) in 3T6 cells. These results indicate the system’s potential for neural cell generation and neural injury recovery research [44].

Carrageenan, a sulfated linear polysaccharide obtained from marine red algae, served as the basis for the construction of a pH-responsive doxorubicin (EPI) delivery system by Chen, Han and their research team. This system was composed of carrageenan oligosaccharide (CAO)-modified gold nanoparticles (AuNPs). CAO-AuNPs showed negligible impact on the viability of normal cells, indicating excellent biocompatibility. EPI-CAO-AuNPs demonstrated superior therapeutic efficacy compared to free EPI, showing reduced cytotoxicity to normal cells while enhancing anticancer activity. The nanoconjugate exhibited significantly lower IC_50_ values for inhibiting HepG2 cells (0.087 ± 0.036 μmol/L) than free EPI (0.173 ± 0.043 μmol/L). Cellular uptake studies revealed efficient endocytosis-mediated internalization and subsequent nuclear localization of released EPI. The formulation induced dose-dependent G2/M phase arrest and apoptosis in both HCT-116 and HepG2 cells more effectively than free EPI. pH-responsive release characteristics were observed, with 96% EPI release at pH 5.0 versus 30% at pH 7.4 within 72 h. This property of enhanced drug release under acidic conditions is advantageous for minimizing the toxicity of EPI to normal tissues [45].

### 6.2. The Treatment of Parasitic Diseases with Seaweed Extract

Nanoparticles, representing a novel and emerging drug delivery system, provide a highly effective strategy for treating parasitic infections. Nanoparticles overcome several challenges associated with antiparasitic drugs, including low bioavailability, poor cellular uptake, nonspecific distribution, and rapid clearance from the body. In recent years, various ideal nanocarriers have been developed to facilitate the delivery of antiparasitic medications [46].

The crude extract of the marine brown alga *Sargassum tenerrimum* was utilized for the green synthesis of silver nanoparticles (Ag-ST) and their antimalarial activity was evaluated. The results indicated that the IC_50_ values of Ag-ST nanoparticles against *P. falciparum* 3D7 and *P. berghei* PB ANKA strains were 7.71 ± 0.39 μg/mL and 23.93 ± 2.27 μg/mL, respectively, both significantly lower than those of silver nanoparticles (Ag-NP) (14.39 ± 1.09 μg/mL and 31.93 ± 1.87 μg/mL), suggesting the superior inhibitory efficacy of Ag-ST against both malaria parasite strains, with further outstanding effects observed in curative and preventive experimental models [47].

The nematicidal activity of extracts from the brown alga *Colpomenia sinuosa*, along with the Ag-NP synthesized from these extracts, has been explored against *Meloidogyne incognita* in vitro. After treating *M. incognita* J2 larvae with the Ag-NP synthesized by *C. sinuosa* at various concentrations for 12, 24, and 72 h, the maximum mortality rates could reach 87%, 98.24%, and 98.28%, respectively, which were comparable to the commercial nematicide Nemacur 400 EC [48].

The series of research advancements outlined above, which have highlighted the utility of marine algal polysaccharides as drug delivery systems and their demonstrated antiparasitic properties, establish a robust foundation for the translational development of terpene-polysaccharide conjugates in parasitic disease treatment. Notwithstanding the absence of reported studies on the direct application of polysaccharide-terpene complexes against parasitic pathogens, the cumulative evidence from optimized carrier systems, validated antiparasitic activities of terpenoid compounds, and nanoparticulate formulations derived from seaweed extracts provides compelling justification for future investigations. These findings collectively suggest that with continued scientific innovation, this interdisciplinary field holds significant promise for breakthroughs in targeted antiparasitic therapies, where polysaccharide-terpene conjugates may emerge as novel therapeutic modalities.

## 7. Discussion

In the research of red algae, studies on the effects of sesquiterpenoids on parasites are relatively common, while in the research of brown algae, more attention is focused on the activity effects of diterpenoids. Among the target parasite species, *Leishmania* has become a key research object largely because it has cell-like characteristics of being passable and cryopreservable. This characteristic provides great convenience for the conduct of relevant research work. Currently, in the field of parasitic disease prevention and treatment research, a crucial question is whether these algal extracts with significant inhibitory effects on *Plasmodium*, *Leishmania*, *Trypanosoma*, etc., can be extended and applied to the treatment of other types of parasites so as to effectively address the increasingly severe problem of parasite drug resistance.

Moreover, judging from the situation of this review, the content related to green algae accounts for a relatively small proportion, which to some extent weakens the persuasiveness of the overall conclusion. Therefore, for relevant researchers, it is necessary to shift the research focus more towards the impact of green algal extracts on parasites and further intensify the comprehensive research on algae, with the hope of discovering more natural drug resources with development potential and providing more options and possibilities for the prevention and treatment of parasitic diseases.

Previous investigations into the antiparasitic properties of algal-derived compounds have primarily focused on terpenoid compounds as the major bioactive constituents. Although numerous studies have reported that various mixtures have different degrees of inhibitory activity against parasites, unfortunately, in these studies, it is often difficult to clearly define which specific components are playing a key role. To elucidate the mechanistic basis of algal-derived compounds’ antiparasitic activity and facilitate their translational development for clinical applications, future research should focus on the meticulous separation of these complex mixtures to obtain compounds with higher purity. On this basis, through in-depth structural analysis, the key chemical groups with antiparasitic activity can be accurately revealed, thereby providing a solid theoretical basis for subsequent industrial synthesis.

In China, the research on seaweed extracts is mainly concentrated in academic institutions in coastal cities. Considering that the central and western regions, such as Sichuan, Qinghai, Xinjiang, Tibet, and Inner Mongolia, have high demands and rich case resources in parasite research, a question worthy of in-depth discussion and planning is how to promote extensive and in-depth cooperation between universities in coastal cities and those in the central and western regions and jointly carry out collaborative research on seaweed extracts in parasite research in these regions. This cross-regional cooperation model is expected to bring new breakthroughs and development opportunities to the research on the treatment of parasitic diseases, further promote the overall progress of China in the field of parasitic disease prevention and treatment, and provide technical support for a community with a shared future for mankind.

## 8. Conclusions

Compared to epidemics, tropical diseases, although receiving relatively low attention, pose a serious threat to public health and the healthy development of the livestock economy. Especially in the treatment of parasitic diseases, there is currently a problem of limited drug types and increased drug resistance. Traditional therapeutic drugs such as ivermectin, artemisinin, albendazole, etc., although effective to some extent, are limited in their long-term use by the emergence of drug resistance. To address this challenge, experts and scholars are actively seeking new therapeutic approaches, with research on natural plant components receiving significant attention. Extracts of terrestrial plants such as allicin, curcumin, resveratrol, and abscisic acid have been proven to have varying degrees of harmful effects on parasites. However, due to the extensive and historical use of terrestrial plant resources, their research scope has become relatively broad, and further exploration may face limitations. In this context, marine algae, as a potential natural medicinal resource, have gradually received attention from researchers in recent years. Marine algae grow in special environments such as high pressure and light avoidance, which may endow them with unique bioactive components. Numerous investigations have demonstrated that bioactive compounds derived from specific marine macroalgae exhibit pronounced antiparasitic efficacy, even surpassing existing drugs. Meanwhile, researchers also conducted in-depth studies on the toxicity of these extracts to ensure their safety. Nevertheless, while marine-derived algal compounds demonstrate considerable promise for parasitic disease management, several critical challenges remain to be resolved. Firstly, we need to gain a deeper understanding of the main chemical structures of these extracts in order to provide a foundation for drug design and synthesis. Secondly, the specific mechanisms by which these compounds resist parasites still need to be elucidated. In addition, considering the limited nature of marine resources, how to effectively utilize algal extract components in industrial production, such as through fermentation or chemical synthesis for large-scale production, is also an important challenge currently faced.

In short, the treatment of parasitic diseases in tropical diseases still needs continuous exploration and innovation. Marine algae, as an emerging natural medicinal resource, have shown broad prospects in the treatment of parasitic diseases. However, to achieve its clinical application and large-scale production, a series of key issues still need to be addressed. In the future, with the deepening of research and advances in technology, we have reason to believe that marine algae extracts will become an important force in the field of parasitic disease treatment.

By searching for dates, it was found that the number of articles on the effects of extracts of marine products such as seaweed, sponges, and microorganisms on parasites has been increasing year by year. These investigations are anticipated to substantially advance the development of novel antiparasitic therapeutics.

## Data Availability

The authors declare that all relevant data supporting the findings of this study are available within the article or from the corresponding authors upon request.

## Abbreviations

*L. donovani*	*Leishmania donovani*
*L. amazonensis*	*Leishmania amazonensis*
*M. infantum*	*Leishmania infantum*
*L. cruzi*	*Leishmania cruzi*
*P. falciparum*	*Plasmodium falciparum*
*S. mansoni*	*Schistosoma mansoni*
*T. cruzi*	*Trypanosoma cruzi*
*T. b. rhodesiense*	*Trypanosoma brucei rhodesiense*
EC_50_	half-maximal effective concentration
IC_50_	half-maximal inhibitory concentration
CC_50_	cytotoxic concentration 50
SI	selectivity index
